# Phosphoproteomics Reveals L1CAM-Associated Signaling Networks in High-Grade Serous Ovarian Carcinoma: Implications for Radioresistance and Tumorigenesis

**DOI:** 10.3390/ijms26104585

**Published:** 2025-05-10

**Authors:** Tihomir Zh Todorov, Ricardo Coelho, Francis Jacob, Viola Heinzelmann-Schwarz, Roger Schibli, Martin Béhé, Jürgen Grünberg, Michal Grzmil

**Affiliations:** 1 Center for Radiopharmaceutical Sciences, PSI Center for Life Sciences, 5232 Villigen PSI, Switzerland; 2 Ovarian Cancer Research, Department of Biomedicine, University Hospital Basel, University of Basel, 4031 Basel, Switzerland; 3 Department of Chemistry and Applied Biosciences, ETH Zurich, 8093 Zurich, Switzerland; 4 Department of Gynecology and Gynecological Oncology, Hospital for Women, University Hospital Basel, 4031 Basel, Switzerland

**Keywords:** phosphoproteomics, L1CAM, radioresistance, ovarian cancer, HGSOC

## Abstract

Quantitative phosphoproteomics enables the comprehensive analysis of signaling pathways driven by overexpressed cancer receptors, revealing the molecular mechanisms that underpin tumor progression and therapy resistance. The glycoprotein L1 cell adhesion molecule (L1CAM) is overexpressed in high-grade serous ovarian carcinoma (HGSOC) and plays a crucial role in carcinogenesis by regulating cancer stem cell properties. Here, CRISPR–Cas9-mediated knockout of *L1CAM* in ovarian cancer OVCAR8 and OVCAR4 cells significantly impaired anchor-independent growth in soft agar assays and reduced clonogenic survival following external beam irradiation. In vivo, *L1CAM* knockout decreased cancer stem cell frequency and significantly decreased tumorigenicity. To uncover L1CAM-regulated signaling networks, we employed quantitative phosphoproteomics and proteomics. Bioinformatics analyses and validation studies revealed L1CAM-associated pathways that contribute to radioresistance through DNA repair processes and mammalian target or rapamycin complex 1 (mTORC1)-mediated signaling. In conclusion, our study established a link between L1CAM-dependent tumorigenesis and radioresistance, both hallmarks of cancer stemness, with phosphorylation of key proteins involved in DNA damage response. This study further emphasizes the value of quantitative phosphoproteomics in cancer research, showcasing its ability to enhance understanding of cancer progression and therapy resistance.

## 1. Introduction

The cell surface glycoprotein L1 cell adhesion molecule (L1CAM) is implicated in tumor progression and therapy resistance across various malignancies, including ovarian cancer (OC), and its overexpression is linked to enhanced cancer stemness and correlates with poor outcomes [1]. In OC patient-derived cancer stem cells (CSCs), L1CAM was found to promote stemness-related properties including sphere formation, tumor initiation, and chemoresistance [2]. A recent study validated L1CAM+/CD133+ cells as ovarian CSCs and identified L1CAM as a potential radioresistance marker and promising therapeutic target [3]. L1CAM has been also recognized as a regulator of cancer stemness in other malignances. In colorectal cancer, L1CAM expression marks a subpopulation of stem-like cells with enhanced tumorigenicity, metastasis-initiating capacity, and chemoresistance [4]. Similarly, in glioblastoma, L1CAM promotes self-renewal and confers temozolomide resistance by maintaining neural stem cell-like properties [5,6], whereas in endometrial cancer, it contributes to the formation of cancer initiating cells and resistance to paclitaxel by activating EMT-associated pathways [7]. These findings suggest that L1CAM plays a conserved role in promoting aggressive, stem-like traits and therapy resistance across multiple malignancies.

Yet, current insight into L1CAM-mediated carcinogenesis and radioresistance, particularly in the most common and lethal type of OC, high-grade serous ovarian carcinoma (HGSOC), remains limited. To uncover L1CAM-regulated signaling pathways, we employed quantitative proteomics and phosphoproteomics, which have become an indispensable tool for a deeper understanding of cancer biology and identification of clinically relevant pathways suitable for therapeutic intervention. This approach is particularly valuable in understanding the complexities of cancer signaling networks and advancing precision medicine for more effective treatment strategies. The clinical relevance of quantitative phosphoproteomics was demonstrated in HGSOC, where an analysis of 169 cancers identified pathways associated with patient outcomes, confirmed homologous recombination deficiency (HRD) status, and informed potential strategies for patient stratification and therapeutic management [8]. Similarly, phosphoproteomics analysis in OC primary cells uncovered kinase signatures, emphasizing the potential of exploring signaling networks to identify druggable pathways [9].

This study revealed L1CAM-regulated oncogenic signaling networks through quantitative phosphoproteomics and showed L1CAM-mediated tumorigenicity and radioresistance in HGSOC models.

## 2. Results

### 2.1. L1CAM Regulates Anchorage-Independent Cell Growth (AICG) and Radioresistance

To investigate the role of L1CAM in ovarian cancer stemness and its contribution to therapy resistance, we generated *L1CAM* knockout (Δ*L1CAM*) HGSOC cells using CRISPR–Cas9 technology. The OVCAR8 and OVCAR4 Cas9+ cells were lentivirally co-transduced with single-guide RNAs (sgRNAs) targeting the extracellular, transmembrane, and intracellular domains of L1CAM (Figure S1), as previously described [10] and outlined in the Materials and Methods section. As controls for CRISPR–Cas9 genome editing, we used sgRNAs targeting the adeno-associated virus integration site 1 (*AAVS1*), a non-essential gene.

**Figure 1:**
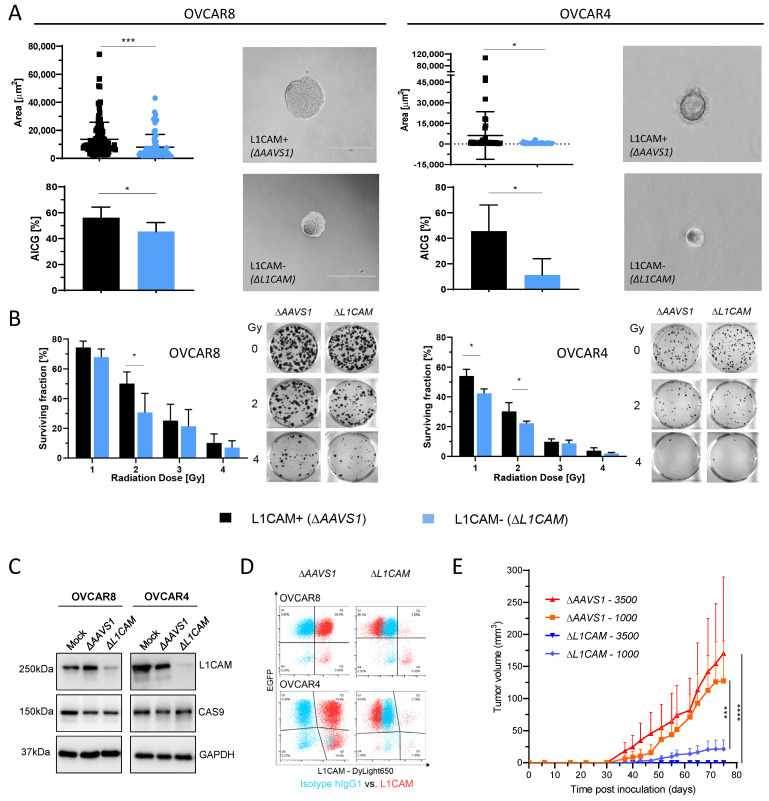
*L1CAM* knockout (Δ*L1CAM*) reduces OC tumorigenicity and radioresistance. (**A**) Surface area of the spheres and anchorage-independent cell growth (AICG) of the single-cell derived colonies presented as percentage (%) of seeded Δ*L1CAM* and control (Δ*AAVS1*) OVCAR8 and OVCAR4 cells. Images show representative spheres 14 days after seeding. Scale bar: 400 µm. (**B**) Surviving fractions shown as % of control and colony images after external beam irradiation. Data indicate mean ± SD (n = 3). (**C**) WB analysis of L1CAM and CAS9 in total protein lysates from control (Mock, Δ*AAVS1*) and Δ*L1CAM* cells. GAPDH was used as a loading control. (**D**) Flow cytometry for L1CAM and EGFP (transfection control). (**E**) Tumor growth curves of xenografted mice inoculated with 1000 and 3500 Δ*AAVS1* or Δ*L1CAM* OVCAR8 cells. Data represent mean tumor volume ± SEM (n = 4). * *p* < 0.05, *** *p* < 0.001, **** *p* < 0.0001.

Loss of L1CAM significantly reduced the AICG and sphere surface area (Figure 1A). In Δ*L1CAM* cells, AICG reached 40–52% and 6–30% as compared to 45–66% and 15–59% in control Δ*AAVS1* OVCAR8 and OVCAR4 cells, respectively. Both Δ*L1CAM* OVCAR8 and OVCAR4 cells showed a significant decrease in clonogenic survival in response to 2 Gy of external radiation as compared to control irradiated cells (Figure 1B). Additionally, 1 Gy resulted in a significant decrease in the clonogenic survival of Δ*L1CAM* OVCAR4 cells. While 3 and 4 Gy exhibited a decreasing survival trend in both knockout (KO) cell lines, the reduction was not statistically significant, likely due to the high overall loss of viability observed in both irradiated control and KO cells. Western blot (WB) analysis (Figure 1C) and flow cytometry (Figure 1D) validated *L1CAM* knockout and expression of EGFP in Cas9-expressing OVCAR8 and OVCAR4 cells. Flow cytometry showed 8.7 ± 1.2% and 5.2 ± 2.5% L1CAM expression in Δ*L1CAM* cells, while the controls (Δ*AAVS1*) expressed L1CAM at 96.5 ± 2.0% and 99.2 ± 0.1% in OVCAR8 and OVCAR4 cells, respectively.

### 2.2. L1CAM-Dependent Tumorigenesis in HGSOC Animal Model

For limiting dilution analysis (LDA) in vivo, we selected the previously validated OVCAR8 cells, which are highly tumorigenic and produced larger tumor masses in comparison to OVCAR4 cells [11]. Control L1CAM-expressing Δ*AAVS1* OVCAR8 cells inoculated at dilutions of 3500 and 1000 cells formed significantly bigger tumors with the lowest latencies in comparison to Δ*L1CAM* cells (Figure 1E, Table S1). Inoculation of 500 cells was a limiting dilution for tumor development (Table S1). *L1CAM* knockout resulted in a 2.3-fold reduction in the cancer stem cell (CSC) frequency, which was sufficient to significantly decrease the tumorigenicity.

### 2.3. Phosphoproteomics Analysis Uncovers L1CAM-Regulated Phosphorylation

**Figure 2:**
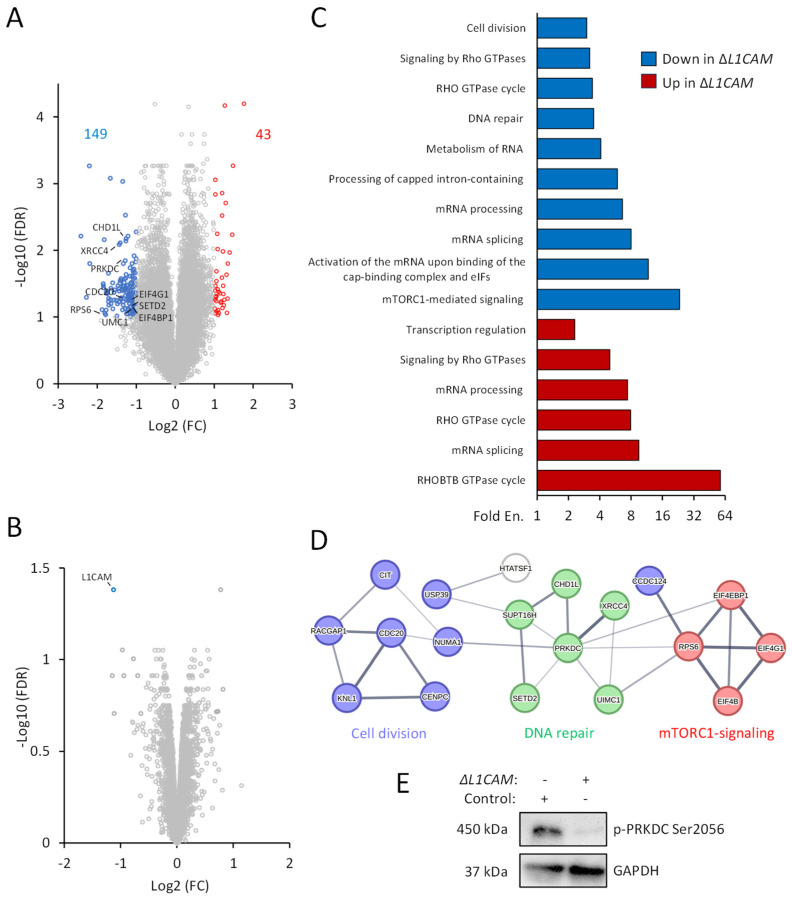
Identification of L1CAM-regulated signaling networks. Protein lysates of Δ*L1CAM* (L1CAM-) and control Δ*AAVS1* (L1CAM+) OVCAR8 cells were subjected to quantitative phosphoproteomics. Volcano plots represent changes of integrated phosphopeptide abundance (**A**) normalized to protein abundance (**B**) in Δ*L1CAM* cells displayed as log2 transformed fold change (FC) in comparison to control cells. Blue and red dots show significant (false discovery rate (FDR), Log10(FDR) > 1) decrease and increase (Log2(FC) ≥|1|) in phosphopeptide or protein abundance, respectively. Examples of phosphopeptides from proteins involved in the cell cycle, DNA repair and mTORC1-mediated signaling are indicated. (**C**) DAVID bioinformatics analysis of the protein groups with down- and up-regulated phosphorylation. Fold Enrichments (Fold En.) for selected biological processes and pathways. The data correspond to the results shown in Table 1. (**D**) Three selected interaction networks of hypophosphorylated proteins in Δ*L1CAM* cells involved in cell division, DNA repair and mTORC1-mediated signaling. The line thickness indicates the strength of interaction confidence. (**E**) WB analysis for PRKDC phosphorylation and GAPDH for loading control in Δ*L1CAM* and control OVCAR8 cells.

To reveal L1CAM-regulated pathways with a focus on radioresistance, we employed quantitative phosphoproteomics and matching proteomics in OVCAR8 cells, where *L1CAM* knockout reduced tumorigenesis in vivo. This analysis identified and quantified the abundance of 24,072 phosphopeptides by phosphoproteomics (Figure 2A) and 7483 protein groups by proteomics analysis (Figure 2B) in Δ*L1CAM* OVCAR8 cells in comparison to Δ*AAVS1* (L1CAM+) OVCAR8 control cells. As expected, decreased L1CAM abundance was identified in Δ*L1CAM* cells. Integration of the proteomics and phosphoproteomics datasets normalized the abundance of 22,100 phosphopeptides to the matching total protein levels and identified 149 and 43 unique phosphopeptides with decreased and increased abundance in Δ*L1CAM* cells, respectively (Figure 2A, Table S2).

### 2.4. Identification of L1CAM-Associated Signaling Networks in HGSOC

Bioinformatics of hypo- or hyperphosphorylated proteins in Δ*L1CAM* cells identified enriched processes including cell division, DNA repair, mammalian target of rapamycin complex 1 (mTORC1)-mediated signaling, activation of the mRNA upon binding of the cap-binding complex and eIFs, or transcription regulation. Both hypo- or hyperphosphorylated proteins influenced mRNA processing, splicing and the Rho GTPase cycle (Figure 2C, Table 1). Interactome analysis visualized networks of hypo- and hyperphosphorylated proteins in Δ*L1CAM* OVCAR8 cells (Figure S2). Further STRING network analysis revealed significant interactions within three selected potential L1CAM-dependent phosphorylated networks associated with radioresistance and tumorigenesis, including cell division, DNA repair and mTORC1-mediated signaling (Figure 2D). WB validation (Figure 2E) in Δ*L1CAM* cells showed decreased S2056 phosphorylation of the protein kinase DNA-activated catalytic subunit (PRKDC).

## 3. Discussion

Our in vitro and in vivo characterization of Δ*L1CAM* models revealed impaired AICG in soft agar assays, reduced clonogenic survival following external beam irradiation, and significantly decreased tumorigenicity. These findings indicate that L1CAM functions as a modulator of cancer stemness in HGSOC, consistent with previous reports of L1CAM-dependent tumorigenesis and radio-chemoresistance in ovarian CSCs [2]. Notably, in the latter studies, transcriptome analysis followed by validation studies identified the L1CAM-regulated FGFR1/SRC/STAT3 signaling pathway in OV90 cells. Furthermore, its expression correlated with the mRNA levels of several stem-cell and EMT-related genes including *LIN28*, *OCT4*, *VIM*, and *TGFB1* in the L1CAM+/CD133+ cell populations derived from ovarian cancer IGROV1 and SKOV3ip cells [3]. Moreover, in the latter study, reintroducing L1CAM expression restored a cancer stem-like phenotype, including clonogenicity, spherogenicity, and radioresistance, in *L1CAM* knockout IGROV1 cells. In a clinical setting, analysis of *L1CAM* mRNA levels in 138 ovarian cancer patients identified L1CAM expression as an independent predictor of disease progression and overall survival, highlighting its significant role in ovarian cancer pathophysiology and its association with poor clinical outcomes [12].

To achieve a broad and comprehensive view of L1CAM downstream signaling, our quantitative phosphoproteomics analysis revealed L1CAM-dependent networks and pathways involved in cell cycle regulation, DNA repair, and mTORC1-mediated signaling. The activation of these pathways provides a mechanistic basis for the impaired tumorigenesis and reduced radioresistance observed in Δ*L1CAM* models. Similar to our findings, in a previous study, *L1CAM* knockdown radiosensitized neuroblastoma cells by simultaneous downregulation of proto-oncogene myelocytomatosis neuroblastoma MycN and the upregulation of tumor suppressor PTEN, which inhibits PI3K/AKT/mTORC1 signaling [13]. In glioblastoma CSCs, L1CAM enhanced DNA damage response (DDR) checkpoints and repair, whereby radiation-induced nuclear translocation of the L1CAM intracellular domain upregulated expression of proto-oncogenes c-Myc and NBS1, which are critical components of the MRE11–RAD50–NBS1 (MRN) complex that activates signaling of ataxia telangiectasia mutated (ATM) kinase [14]. In ovarian cancers, including HGSOC, the PI3K/AKT/mTOR pathway is frequently hyperactivated through various genetic alterations, driving cell proliferation, migration, and chemotherapy resistance [15,16]. This suggests that L1CAM-associated mTORC1 signaling contributes to the tumorigenic properties of L1CAM, yet this point warrants further investigation.

In the present study, phosphoproteomics and WB validation in Δ*L1CAM* cells showed decreased S2624 and S2056 phosphorylation, respectively, of protein kinase DNA-activated catalytic subunit (PRKDC), also known as DNA-dependent protein kinase catalytic subunit (DNAPKC), a key mediator of non-homologous end-joining (NHEJ) during the response to DNA damage. Both S2624 and S2056 are in the N-terminal domain of DNAPKC in the ABCDE and PQR clusters, respectively, whose phosphorylation regulates DNAPKC activity toward DNA end ligation and resection during DNA double strand break (DDB) repair [17]. Furthermore, Δ*L1CAM* cells had decreased phosphorylation of ribosomal protein S6 (RPS6) at S235/S236 and eukaryotic translation initiation factor 4E-binding protein 1 (EIF4EBP1) at S65, well-characterized downstream substrates and markers for activated mTORC1, which regulates cell metabolism, protein and lipid synthesis, proliferation, and survival, leading to increased cancer progression and therapy resistance [18,19]. In a previous study, pharmacological inhibition of the mTOR pathway using the dual PI3K/mTOR inhibitor LY3023414 induced DNA damage and enhanced CHK1 inhibitor-induced replication stress and cancer cell death in HGSOC models, including OVCAR8 and PEO1 cells [20]. Similarly, the mTOR inhibitor INK128 re-sensitized ovarian cancer cells to carboplatin-induced DNA damage by disrupting the selective translation of mRNAs involved in DNA repair and cell survival [21], highlighting the critical role of mTOR signaling in mediating resistance to DNA damage-based therapies. Combining inhibition of mTORC1 or DNAPKC signaling with radio-chemotherapy has shown promising results in cancer therapy [17,18], suggesting that targeting L1CAM, which influences DNA repair and mTORC1 signaling, could be a valuable approach for further therapeutic exploration. Other L1CAM-influenced cellular processes such as RNA processing, splicing and the RHO GTPase cycle have also been associated with malignant transformation cancer stemness and radioresistance [22,23]. Yet, this point warrants further investigation. In conclusion, our study linked L1CAM-dependent tumorigenesis and radioresistance, both hallmarks of cancer stemness, with phosphorylation of key proteins involved in DNA repair and mTORC1 signaling in HGSOC cells.

## 4. Materials and Methods

### 4.1. Cell Culture and Generation of ΔL1CAM Knockout Cells

Ovarian cancer OVCAR8 and OVCAR4 cells were kindly provided by Viola Heinzelmann-Schwarz and Francis Jacob (University Hospital Basel (USB), Basel, Switzerland) and cultured in RPMI 1640 medium containing 10% FCS, 100 U/mL penicillin, 0.1 mg/mL streptomycin and 1% L-glutamine. All cells were short tandem repeat (STR) authenticated (Microsynth, Balgach, Switzerland) and cultured at 37 °C at 5% CO_2_ in a humidified incubator. The knockout (Δ*L1CAM*) and control (Δ*AAVS1*) cells were generated using CRISPR–Cas9 gene-editing technology as described below and maintained in growth medium supplemented with 1 µg/mL puromycin.

### 4.2. Generation of ΔL1CAM Knockout Cells

For molecular cloning, single guide RNAs (sgRNAs) with targeting *L1CAM* protein-coding genome DNA sequences were designed using the guide design tool Benchling Biology Software, 2022, https://benchling.com (accessed on 1 July 2022). SgRNAs with high on-target and off-target scores for *L1CAM* exon 2 (CCTGCTTATCCAGATCCCCG), exon 4 (TGACATCAGCCTCAAGTGTG), exon 25 (TGGTTGTAGCTGACATACTG), and exon 26 (CAGTGGCGAAGCCAGCAGGA) were selected with the following protospacer adjacent motif (PAM) sequences: AGG, AGG, TGG and GGG, respectively. Single-strand oligonucleotides (Sigma-Aldrich, Buchs, Switzerland) were cloned into the LRG2.1 plasmid (Addgene, #108098, Watertown, MA, USA) using the BsmBI endonuclease restriction site. Annealed and phosphorylated oligonucleotides were ligated into the plasmid with T4 DNA ligase (Promega, Dübendorf, Switzerland). For amplification, the ligations were transformed into Stbl3 *E. coli* via heat shock following ampicillin selection and Plasmid Miniprep (ZYMO Research, Lucerna-Chem AG, Luzern, Switzerland). Sanger DNA Sequencing (Microsynth, Balgach, Switzerland) was performed to confirm the insertion of the sgRNAs into the plasmid in the human U6 promoter (5′-GAG GGC CTA TTT CCC ATG ATT-3′). For lentivirus production, human embryonic kidney HEK293T cells were seeded at 50% confluency in 75 cm^2^ tissue culture flasks one day before transfection. A total of 4 μg of LRG2.1 (Addgene #108098) or LRG2.1_mOrange (Addgene #124772) encoding the sgRNA of interest or LentV-Cas9-puro (Addgene #108100), 2 μg of pMD2.G (Addgene #12259), and 2 μg of pCMVR8.74 (Addgene #22036) were co-transfected using 24 μL of jetPEI reagent in 1 mL of 150 mM NaCl solution (Polyplus-transfection). The growth medium was changed 24 h after transfection. Supernatant containing lentivirus particles was collected 48 h later and filtered with a 0.45 μm polyvinylidene fluoride filter (Sartorius AG, Göttingen, Germany), aliquoted into 1.5 mL cryotubes, and stored at −80 °C until further use. Transduced cells were selected with 1–3 µg/mL puromycin for 1 week. Selected Cas9+ cell lines were kept in media containing 1 µg/mL puromycin. To generate Δ*L1CAM* and control Δ*AAVS1* cells, constitutively expressing Cas9 cells were checked by flow cytometry for L1CAM expression and seeded at 2 × 10^5^ cells per well on 6-well plates in culture medium under 1 µg/mL puromycin selection. On the next day, the lentiviral supernatants containing viral particles targeting the selected *L1CAM* exons or *AAVS1* were used for transduction. After 24 h, the media was exchanged and the cells were incubated for two more days before expansion. All cell lines underwent EGFP-enrichment via fluorescence activated cell sorting (FACS, BDFACSaria Cell Sorter, BD Bioscience, Franklin Lakes, NJ, USA) and were further expanded.

### 4.3. Anchorage-Independent Cell Growth (AICG) and Radiation Response Assay

Cells were resuspended in medium containing 0.4% low melt agarose (Sigma-Aldrich) and seeded at a cell density of 1000 cell per well in 6-well plates coated with 0.6% low melt agarose. Cells were incubated for 14 days in 2 mL culture medium prior to imaging (Primovert, Zeiss, Oberkochen, Germany) and colony analysis with Fiji software (Fiji/ImageJ version 2.14.0) [24]. AICG % was expressed as the ratio of the number of formed colonies to the total number of seeded cells. To assess radiation response, irradiated cells were subjected to imaging and survival fraction determination as described in the Supplementary Materials. Statistical analyses were performed with GraphPad Prism 8.3.1, and unpaired two-tailed *t*-tests were used for both assays.

### 4.4. Western Blot (WB) Analysis

Antibodies against L1CAM (chCE7) were produced at Paul Scherer Institute, (PSI) [3], whereas anti-Cas9 (7A9-3A3), anti-phospho-DNA-PKCS Ser2056 (68716), and anti-GAPDH (14C10) antibodies were from Cell Signaling Technology (CST, Allschwil, Switzerland) and Santa Cruz Biotechnology (SCBT, Heidelberg, Germany), respectively. Total protein lysates were prepared in RIPA buffer supplemented with protease inhibitor cocktail (Roche, Basel, Switzerland) and subjected to standard WB using SDS–PAGE separation, PVDF membranes (Millipore, Sigma-Aldrich, Buchs, Switzerland) blocked with 5% skim milk in TBST (0.1% Tween 20) for 1 h and incubated with 2% BSA in TBST overnight with the primary antibody followed by 2 h incubation with anti-rabbit or anti-mouse HRP-conjugated secondary antibody (CST). Protein-specific signals were detected by a chemiluminescence reagent (ECL) and acquired using an ImageQuant RT ECL Imager (GE Healthcare, Chicago, IL, USA).

### 4.5. Flow Cytometry

Cells were detached in non-enzymatic cell dissociation solution (Sigma-Aldrich), stained with anti-L1CAM mAb chCE7 (50 ng/µL, produced at PSI) or control IgG1 (50 ng/µL, # 02-7102, Invivogen, Thermo Fisher Scientific, Waltham, MA, USA) and then with a secondary anti-IgG1 antibody (Goat F(ab’)2 anti-human IgG—Fc, pre-adsorbed, DyLight650-conjugated, ab98593, Abcam, Cambridge, UK) followed by DAPI staining (0.1 ng/µL, BioLegend, San Diego, CA, USA) for 5 min. All steps were performed in 100 µL of FACS buffer (1% FCS in PBS) for 30 min on ice in the dark, and the cells were washed twice with FACS buffer after each step. All cell lines were gated to exclude cellular debris, doublets and dead cells (DAPI+), and according to the isotype controls to determine the antigen positive cells.

### 4.6. Limiting Dilution Analysis (LDA)

Δ*L1CAM* or Δ*AAVS1* OVCAR8 cells were mixed in RPMI supplemented with 1% L-Glutamine:ECM gel (E6909, Sigma-Aldrich) in a 1:1 ratio. Subsequently, 500, 1000 or 3500 cells were inoculated subcutaneously into the right and left flanks of CD-1 (Crl:CD1-Foxn1nu) nude mice (Charles River, Sulzfeld, Germany, 4–5-week-old females). The tumor volume was non-invasively measured with a caliper twice a week for 140 days and the animals were euthanized if the tumors exceeded 1 cm^3^ or started to ulcerate. Two-way ANOVA with Turkey’s multiple comparisons was used for statistical analysis, whereas ELDA software (https://bioinf.wehi.edu.au/software/elda/, accessed on 29 November 2023) [25] was used to determine the CSC frequency.

### 4.7. Preparation of Tryptic Peptides and Phosphopeptide Enrichment

Cells grown on 60 cm^2^ petri dishes at 90% confluence were detached with accutase (Sigma-Aldrich, Buchs, Switzerland), washed with ice-cold PBS, snap-frozen in liquid nitrogen, and stored at −80 °C. The cell pellets (three biological replicates per condition) were lysed in 100 µL 4% SDS/Tris-HCl, pH 8.5 and protein extraction was carried out using a tissue homogenizer (TissueLyser II, QUIAGEN, Hilden, Germany) by applying 2 × 2 min cycles at 30 Hz. In addition, the samples were boiled for 10 min at 95 °C, followed by two rounds of High Intensity Focused Ultrasound (HIFU) for 1 min each at an ultrasonic amplitude of 100%. The samples were treated with 5 units of benzonase for 15 min at 30 °C before centrifugation at 20,000× *g* for 10 min. The protein concentration was estimated using a Lunatic UV/Vis polychromatic spectrophotometer (Unchained Labs, Pleasanton, CA, USA) and the samples were diluted 1:20. For each sample, 100 µg of protein was used for protein digestion following a modified version of the filter-aided sample preparation (FASP) protocol [26]. Briefly, proteins were diluted in 200 µL of 8 M urea in 100 mM Tris/HCL pH 8.2 (UT) buffer, loaded on an Ultracel 30,000 molecular weight cutoff (MWCO) centrifugal unit (Amicon Ultra, Merck, Rahway, NJ, USA) and centrifuged at 14,000× *g*. The SDS buffer was exchanged by one centrifugation round of 200 µL UT buffer. Alkylation of reduced proteins was carried out by 5 min incubation with 100 µL 0.05 M iodoacetamide in UT buffer, followed by three 100 µL washing steps with UT and two 100 µL washing steps with triethylammonium bicarbonate buffer (TEAB, pH 8). Finally, proteins were on-filter digested using 120 µL of 0.05 M TEAB (pH 8) containing trypsin (Promega, Madison, WI, USA) at a ratio of 1:50 (*w*/*w*). Digestion was performed overnight in a wet chamber at RT. Peptides were eluted by centrifugation at 14,000× *g* for 20 min and dried to completeness.

The phosphopeptide enrichment was performed using a KingFisher Flex System (Thermo Fisher Scientific) and Ti-IMAC HP MagBeads (ReSyn Biosciences, Gauteng, NA, USA). Beads were conditioned following the manufacturer’s instructions, consisting of 3 washes with 200 µL of binding buffer (80% acetonitrile, 5% TFA, 0.1 M glycolic acid). Each fraction was dissolved in 200 µL binding buffer and an aliquot of 2 µg per sample was taken for the whole proteome analysis. The beads, wash solutions and samples were loaded into 96 well microplates and transferred to the KingFisher. Phosphopeptide enrichment was carried out using the following steps: washing of the magnetic beads in binding buffer (5 min), binding of the phosphopeptides to the beads (30 min), washing the beads in wash 1 and 2 (wash buffer 1: 80% acetonitrile, 1% TFA; wash buffer 2: 10% acetonitrile, 0.2% TFA; 3 min each) and peptide elution from the beads (80 µL 1% NH_4_OH in water, 10 min). To each elution, 10 µL of 10% formic acid was added. The enriched as well as the full proteome samples were loaded onto Evotips according to the manufacturer’s instructions.

### 4.8. Tandem Mass Tag (TMT) Labeling and Peptide Fractionation

Half of each sample was used for TMT labeling. Two hundred and fifty µg TMT 18-plex reagent (Thermo Fisher Scientific) was dissolved in 15 μL of anhydrous acetonitrile (Sigma-Aldrich, Buchs, Switzerland) and added to 50 µg peptides in 45 µL of 50 mM TEAB, pH 8.5. The solution was gently mixed and incubated for 60 min at RT. The reaction was quenched by adding 3.5 µL of 5% hydroxylamine (Thermo Fisher Scientific). The combined TMT sample was created by mixing equal amounts of each TMT channel together. Labeled peptides were offline pre-fractionated using high pH reverse-phase chromatography. Peptides were separated on an XBridge Peptide BEH C18 column (130 Å, 3.5 µm, 1.0 mm × 250 mm, Waters, Milford, MA, USA) using a 72 min linear gradient from 5–40% acetonitrile/9 mM NH_4_HCO_2_. Every minute, a new fraction was collected and concatenated into 36 final fractions. The fractions were dried.

Liquid chromatography with tandem mass spectrometry (LC-MS/MS) analysis MS analysis was performed on an Orbitrap Exploris 480 mass spectrometer (Thermo Fisher Scientific) equipped with a Flex source (Thermo Fisher Scientific) and coupled to an Evosep One (Evosep, Odense, Denmark). The solvent composition of the two channels was 0.1% formic acid for channel A and 0.1% formic acid, 99.9% acetonitrile for channel B. The column temperature was 50 °C. Peptides were separated on a commercial PepSep C18 Column (3 µm, 150 µm × 15 cm, Bruker, Billerica, MA, USA) using the 30SPD method. The MS was operated in data-dependent mode (DDA) with a maximum cycle time of 3 s, funnel RF level at 40% and heated capillary temperature at 275 °C. Full-scan MS spectra (350−1500 *m*/*z* for proteome samples and 350–1800 *m*/*z* for enriched samples) were acquired at a resolution of 120,000 at 200 *m*/*z* after accumulation to a target value of 3,000,000 or for a maximum injection time of 45 ms. Precursors with an intensity above 2000 were selected for MS/MS. Ions were isolated using a quadrupole mass filter with a 0.7 *m*/*z* isolation window and fragmented by higher-energy collisional dissociation (HCD) using a normalized collision energy of 32%. HCD spectra were acquired at a resolution of 45,000 and the maximum injection time was set to Auto for proteome samples and to 200 ms for enriched samples. The normalized automatic gain control (AGC) was set to 100%. Charge state screening was enabled such that singly, unassigned and charge states higher than seven were rejected. Precursor masses previously selected for MS/MS measurement were excluded from further selection for 8 s, and the exclusion window was set at 10 ppm. The samples were acquired using internal lock mass calibration on *m*/*z* 371.1012 and 445.1200. The MS proteomics data were handled using the local laboratory information management system (LIMS) [27].

### 4.9. Proteomics and Phosphoproteomics Data Analysis and Statistics

The acquired shotgun MS data were processed for identification and quantification using Fragpipe 19.0 (Philosopher 4.8.1). Spectra were searched against a concatenated Uniprot human reference proteome and Uniprot bovine reference proteome (reviewed canonical version from 17 October 2023 concatenated to its reversed decoyed fasta database and common protein contaminants) using MSFragger 3.5 and Percolator. TMT modification on peptide N-termini and Lysine side chains as well as carbamidomethylation of cysteine were set as a fixed modification, while methionine oxidation was set as a variable. Enzyme specificity was set to trypsin/P, allowing a minimal peptide length of 7 amino acids and a maximum of two missed cleavages. Reporter ion intensities were extracted with 20 ppm integration tolerance. For peptide and protein quantification, the co-isolation filter was set to 50%.

For statistical evaluation of the total proteome dataset, the FragPipe output “psm.tsv” was used as input to the prolfqua R-package [28]. In brief, a minimum abundance of 1 was required for each TMT-channel and otherwise it was filtered out, then the TMT abundances for all PSMs were aggregated for all PSMs of the same protein using the median-polish method. This protein abundance was then log2-transformed and normalized with a robust z-score transformation. A linear model was fitted to each protein abundance estimate to compute significant differences between different conditions. These group comparisons (contrasts) were evaluated with a moderated Wald-test with pooled variance (as implemented in the limma R-package [29]. The resulting *p*-values were adjusted for multiple testing using the Benjamini-Hochberg procedure (BH). Missing values did not exist and therefore did not need to be handled. For the phospho-enriched dataset, the TMT-report “abundance_multi-site_None.tsv” was used. These TMT-integrator reports are prefiltered with a best localization probability at a minimum 0.75. The index column of this file concatenated with the peptide sequence served as an identifier for each phospho-peptide. Phosphosite-centric TMT abundances (default median aggregation from the PSM level) were log2-transformed and normalized, fitted and contrasted as described above for the total proteome dataset. After assessing differential expression on the two datasets individually, the results of the total proteome analysis were joined with the phosphosite-centric results. Contaminant proteins were filtered out, and for each phospho-peptide assignment, the number of phosphorylations, the modified residue, and its position in the protein were parsed. The estimated log2FC for the phosphosites were adjusted for the change that was estimated on the protein level using the procedure suggested by Kohler et al. [30] using equations 9–12. This adjusted log2FC and new *p*-value was again adjusted for multiple testing using BH. The mass spectrometry proteomics data have been deposited at the ProteomeXchange Consortium via the PRIDE [31] partner repository.

### 4.10. Bioinformatics Analysis

Identified phosphoproteins were analyzed using the Database for Annotation, Visualization and Integrated Discovery (DAVID) bioinformatics platform v2023q4 [32]. Fold Enrichment was scored for terms representing biological processes (GOTERM_BP DIRECT) and signaling pathways (REACTOME_PATHWAY) with *p* < 0.05 and False Discovery Rate (FDR) ≤ 0.1 for a group of at least three proteins. The STRING database [33] (version 12.0) was used to visualize protein–protein associations based on active interaction sources (score > 0.4). Disconnected nodes were excluded from the network.

## 5. Conclusions

This first phosphoproteomics study of *L1CAM* knockout in high-grade serous ovarian carcinoma cells links L1CAM-dependent tumorigenesis and radioresistance, both hallmarks of cancer stemness, with phosphorylation of key proteins involved in DNA repair and mTORC1 signaling. Collectively, our findings together with previous studies indicate that human L1CAM has the potential to serve as a CSC marker and as a therapeutic target for cancer treatment.

## Figures and Tables

**Table 1:** Significantly enriched biological processes and pathways in OVCAR8 Δ*L1CAM* cells identified by DAVID bioinformatics analysis. Fold Enrichment (Fold Enr.) was scored for functional annotation terms (UP_KW_BIOLOGICAL_PROCESS and REACTOME_PATHWAY) with *p* < 0.05 and False Discovery Rate (FDR) ≤ 0.1 for a group of at least three proteins.

Terms and Identified Hypo-Phosphorylated Proteins	Protein No.	Fold. Enr.	*p*	FDR
**DNA repair**				
HTATSF1, SETD2, SUPT16H, XRCC4, BOD1L1, CHD1L, NUCKS1, PRKDC, UIMC1 (UP_KW_BIOLOGICAL_PROCESS)	9	3.5	4.1 × 10^−3^	6.2 × 10^−2^
**mTORC1-mediated signaling**				
EIF4B, EIF4EBP1, EIF4G1, RPS6 (REACTOME_PATHWAY)	4	23.4	6.1 × 10^−4^	3.3 × 10^−2^
**Metabolism of RNA**				
ACIN1, CPSF7, CWC22, DHX38, EIF4B, EIF4G1, HNRNPD, HTATSF1, NCL, NOP2, RBM28, RBMX, RPS28, RPS6, SMN1, SRRM2, SRSF4, SRSF5, TRA2B, TRMT6, USP39 (REACTOME_PATHWAY)	21	4.1	8.6 × 10^−8^	3.2 × 10^−5^
**mRNA processing**				
BCLAF1, CWC22, DHX38, HTATSF1, RBM28, RBMX, ACIN1, CPSF7, PRPF38B, SRSF4, SRSF5, SRRM2, SREK1, SMN1, THRAP3, TRA2A, TRA2B, USP39, ZC3H13 (UP_KW_BIOLOGICAL_PROCESS)	19	6.6	2.8 × 10^−10^	6.3 × 10^−9^
**Processing of capped intron-containing pre-mRNA**				
ACIN1, CPSF7, CWC22, DHX38, HNRNPD, HTATSF1, RBMX, SRRM2, SRSF4, SRSF5, TRA2B, USP39 (REACTOME_PATHWAY)	12	5.9	4.5 × 10^−6^	4.2 × 10^−4^
**mRNA Splicing**				
ACIN1, BCLAF1, CWC22, DHX38, HTATSF1, PRPF38B, RBM28, RBMX, SMN1, SREK1, SRRM2, SRSF4, SRSF5, THRAP3, TRA2A, TRA2B, USP39, ZC3H13 (UP_KW_BIOLOGICAL_PROCESS)	18	8	4.6 × 10^−11^	2.0 × 10^−9^
**Activation of the mRNA upon binding of the cap-binding complex and eIFs, and subsequent binding to 43S**				
EIF4B, EIF4EBP1, EIF4G1, RPS28, RPS6 (REACTOME_PATHWAY)	5	11.7	8.2 × 10^−4^	3.8 × 10^−2^
**Signaling by Rho GTPases**				
AKAP12, CDC20, CDC42BPA, CDC42EP1, CENPC, CIT, CLIP1, CPSF7, CTTN, DOCK5, DOCK7, DST, KNL1, RACGAP1, RBMX, TRA2B (REACTOME_PATHWAY)	16	3.2	1.0 × 10^−4^	7.5 × 10^−3^
**RHO GTPase cycle**				
AKAP12, CDC42BPA, CDC42EP1, CIT, CPSF7, DOCK5, DOCK7, DST, RACGAP1, RBMX, TRA2B (REACTOME_PATHWAY)	11	3.4	1.1 × 10^−3^	4.8 × 10^−2^
**Cell division**				
CCDC124, CDC20, CENPC, CIT, KNL1, NUMA1, RACGAP1, USP39, ZC3HC1 (UP_KW_BIOLOGICAL_PROCESS)	9	3.0	9.3 × 10^−3^	1.0 × 10^−1^
**Terms and Identified Hyper-Phosphorylated Proteins**	**Protein No.**	**Fold. Enr.**	***p***	**FDR**
**Transcription regulation**				
ESF1, HTATSF1, MAX, NIPBL, SAP30BP, SUB1, EEF1D, IWS1, SALL2, TCEAL4, ZBTB7A (UP_KW_BIOLOGICAL_PROCESS)	11	2.3	9.0 × 10^−3^	6.3 × 10^−2^
**mRNA processing**				
HTATSF1, GEMIN5, HNRNPC, IWS1, TRA2A, TRA2B (UP_KW_BIOLOGICAL_PROCESS)	6	7.4	8.5 × 10^−4^	9.7 × 10^−3^
**mRNA splicing**				
HTATSF1, GEMIN5, HNRNPC, IWS1, TRA2A, TRA2B (REACTOME_PATHWAY)	6	9.5	2.7 × 10^−4^	6.1 × 10^−3^
**RHO GTPase cycle**				
AKAP12, TIAM2, HSP90AA1, HSP90AB1, HNRNPC, NUDC, TRA2B (REACTOME_PATHWAY)	7	7.9	1.4 × 10^−4^	9.4 × 10^−3^
**Signaling by Rho GTPases**				
AKAP12, TIAM2, HSP90AA1, HSP90AB1, HNRNPC, NUDC, TRA2B (REACTOME_PATHWAY)	7	5	1.5 × 10^−3^	7.1 × 10^−2^
**RHOBTB GTPase cycle**				
HSP90AA1, HSP90AB1, HNRNPC, TRA2B (REACTOME_PATHWAY)	4	57.6	3.7 × 10^−5^	3.8 × 10^−3^

## Data Availability

The mass spectrometry proteomics data have been deposited at the ProteomeXchange Consortium with dataset identifier PXD055521. Other generated data are available in the Supplementary Material.

## Supplementary Materials

The following supporting information can be downloaded at: https://www.mdpi.com/article/10.3390/ijms26104585/s1.

## Abbreviations

AICG	Anchorage-independent cell growth
CSCs	Cancer stem cells
DDR	DNA damage response
DNAPKC	DNA-dependent protein kinase catalytic subunit
HGSOC	High-grade serous ovarian carcinoma
L1CAM	L1 cell adhesion molecule
LDA	Limiting dilution analysis
mTORC1	Mammalian target of rapamycin complex 1
NHEJ	Non-homologous end-joining
OC	Ovarian cancer
PRKDC	Protein kinase DNA-activated catalytic subunit
TMT	Tandem mass tag
