# Supplementary material for: Phosphoproteomics Reveals L1CAM-Associated Signaling Networks in High-Grade Serous Ovarian Carcinoma: Implications for Radioresistance and Tumorigenesis
Source: Int J Mol Sci. 2025 May 10;26(10):4585. doi: 10.3390/ijms26104585 (PMC12111665; doi:10.3390/ijms26104585)
Supplement: Supplementary file 1 [file ijms-26-04585-s001.zip › ijms-3570983-supplementary.pdf]

Supplementary Material

Phosphoproteomics Reveals L1CAM-Associated Signaling Networks in High-Grade Serous Ovarian Carcinoma: Implications for Radioresistance and Tumorigenesis

Tihomir Zh Todorov[1,2,3], Ricardo Coelho[2], Francis Jacob[2], Viola Heinzelmann-Schwarz[2,4], Roger Schibli[1,3], Martin Béhé[1], Jürgen Grünberg[1] and Michal Grzmil[1,*]

[1]*Center for Radiopharmaceutical Sciences, PSI Center for Life Sciences, 5232 Villigen PSI, Switzerland*

[2]*Ovarian Cancer Research, Department of Biomedicine, University Hospital Basel, University of Basel, 4031 Basel, Switzerland*

[3]*Department of Chemistry and Applied Biosciences, ETH Zurich, 8093 Zurich, Switzerland*

[4]*Department of Gynecology and Gynecological Oncology, Hospital for Women, University Hospital Basel, 4031 Basel, Switzerland*

* Correspondence: michal.grzmil@psi.ch

Supplementary Figure S1 and S2
Supplementary Table S1 and S2

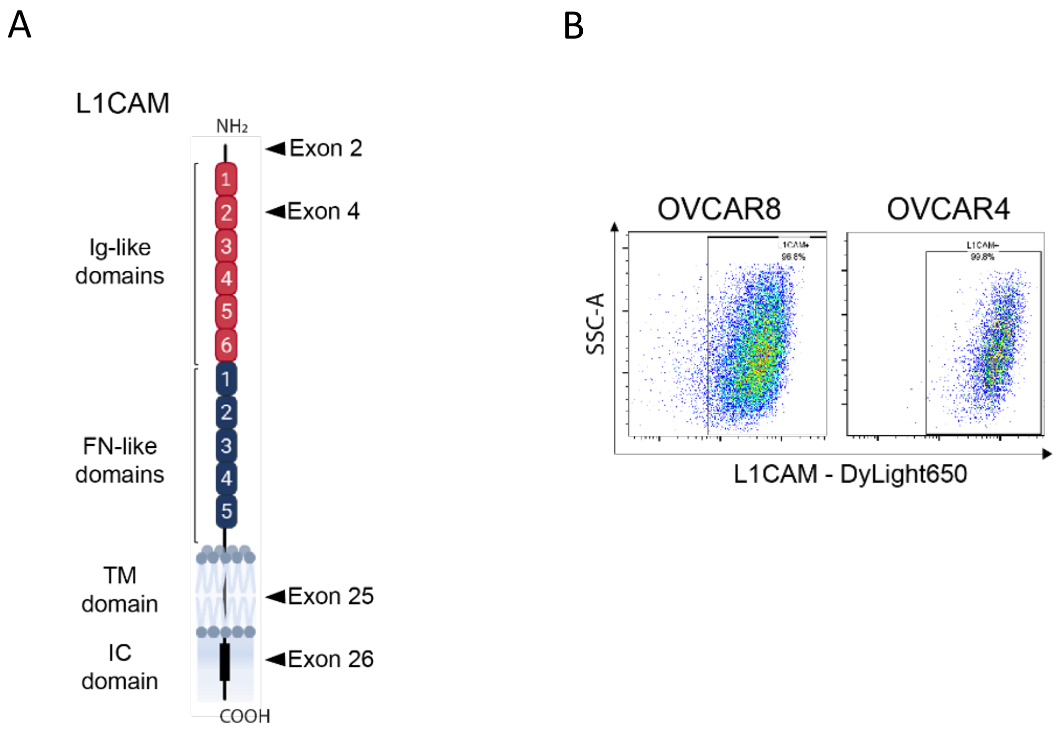

Supplementary Figure S1. Schematic representation of L1CAM domains and its expression in ovarian cancer cells. (A) L1CAM protein domain structure showing the protein regions encoded by the exons targeted with sgRNAs (arrows) for CRISPR-*Cas9* pool KO. (B) Representative flow cytometry analysis for L1CAM in OVCAR8 and OVCAR4 cells with constitutive *Cas9* expression. Ig, human immunoglobulin; FN, fibronectin; TM, transmembrane; IC, intracellular.

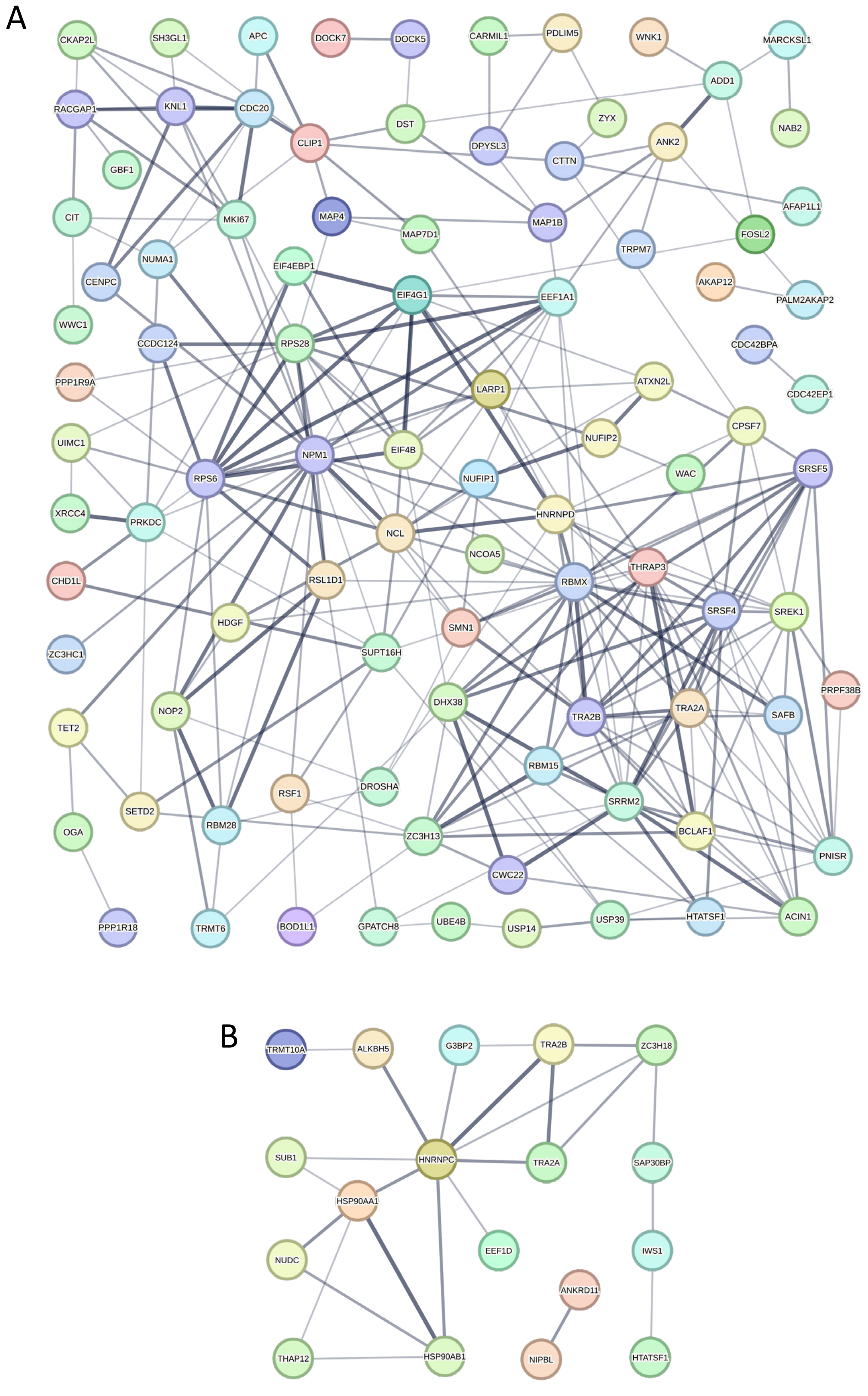

Supplementary Figure S2. Interaction networks of identified hypophosphorylated (**A**) and hyperphosphorylated proteins (**B**) in Δ*L1CAM* OVCAR8 cells. The line thickness indicates the strength of interaction confidence.

Supplementary Table S1. Tumor formation and latency. The CSC frequency was calculated with the Extreme Limiting Dilution Analysis for stem cell research software (ELDA, https://bioinf.wehi.edu.au/software/elda/) with 95% confidence interval. d, days

	OVCAR8 L1CAM+ (*ΔAAVS1*)		OVCAR8 L1CAM- (*ΔL1CAM*)	
Cell dose	Tumor formation	Tumor latency (d)	Tumor formation	Tumor latency (d)
3 500	5 / 8	30 - 43	2 / 8	57
1 000	5 / 8	30 - 51	3 / 8	37 - 51
500	0 / 8	-	1 / 8	37 - 43
CSC frequency	1 / 2 674		1 / 6 005	

Supplementary Table S2. Significantly altered phosphopeptide abundance normalized to the total protein level in OVCAR8 *ΔL1CAM* versus control OVCAR8 *ΔAAVS1* cells. Integrated phosphoproteomics and proteomics data. Log2 fold change (FC) ≥ I1I, False Discovery Rate (FDR) < 0.1. *Phosphopeptide abundance not normalized to the total protein levels due to missing corresponding total protein data.

Symbol	Uniprot ID_ Sequence window position	FC Log2	FDR -Log10
CTTN	Q14247_438_453_1_1_Y446~GPVSGTEPEPVySMEAADYR	-2.42	2.21
RBMX	P38159_116_124_1_1_S116~GGsGGTRGPPSR	-2.28	1.30
GPATCH8	Q9UKJ3_1033_1038_2_2_S1033S1035~sQsPHYFR	-2.21	3.27
CTTN	Q14247_438_453_1_1_S447~GPVSGTEPEPVYsMEAADYR	-2.19	1.80
BCLAF1	Q9NYF8_881_888_1_1_S884~SGSsPKWTHDK	-1.88	1.11
RBM15	Q96T37_765_781_1_0~LKSPSQKQDGGTAPVASASPK	-1.85	1.50
EMILIN2	Q9BXX0_644_644_1_1_T644~AGENAGMGRFtKVGEQER*	-1.85	1.47
SMN1	Q16637_62_69_1_0~NGDICETSGKPKTTPK	-1.84	1.44
CTTN	Q14247_438_453_1_0~GPVSGTEPEPVYSMEAADYR	-1.82	2.16
RPS6	P62753_235_242_2_2_S235S236~RLssLRASTSK	-1.80	1.06
CASP4	P49662_271_292_1_1_S274~DSPAsLEVASSQSSENLEEDAVYK	-1.79	1.04
RACGAP1	Q9H0H5_588_595_1_1_S592~TPSSsSLSQR	-1.77	1.10
SRRM2	Q9UQ35_846_866_2_2_T848T866~SGtPPRQGSITSPQANEQSVtPQRR	-1.77	1.37
CIT	O14578_1993_2002_1_1_S1993~ERsPGRLFEDSSR	-1.76	1.39
NCL	P19338_563_563_1_1_S563~LELQGPRGsPNAR	-1.74	1.35
CHCHD3	Q9NX63_46_58_1_1_S50~SQRYsGAYGASVSDEELK	-1.72	1.23
WWC1	Q8IX03_833_841_1_1_T835~SStQTLEDSWR	-1.71	1.66
NUFIP1	Q9UHK0_292_292_1_1_S292~IRsPGKNHK	-1.71	1.28
CAP2	P40123_299_312_2_1_S301~AQGGQTQsPTKSHTPSPTSPK	-1.70	1.18
NCOA5	Q9HCD5_29_34_1_1_S29~DRsPIRGSPR	-1.67	1.26
SREK1	Q8WXA9_435_437_1_1_S435~ERsTSMR	-1.67	3.08
WNK1	Q9H4A3_2270_2276_1_0~RGSKGHMNYEGPGMAR	-1.65	1.43
BCLAF1	Q9NYF8_881_888_1_0~SGSSPKWTHDK	-1.65	1.20
BOD1L1	Q8NFC6_1462_1462_1_1_S1462~HKRsPGK	-1.61	1.51
TMEM245	Q9H330_12_16_1_1_S16~DAPSLRSsPGPAPR	-1.60	1.12
OGA	O60502_6_9_1_1_T9~ESQAtLEER	-1.57	1.25
DOCK5	Q9H7D0_1824_1835_1_1_S1834~SKPYEGSQRNsTELAPPLPVR	-1.57	1.37
MXRA7	P84157_143_144_1_1_S144~YsPGKLR	-1.55	1.41
SRRM2	Q9UQ35_1818_1831_3_3_S1818Y1820S1822~GGsGyHsRSPARQESSR	-1.51	1.25
ZC3H13	Q5T200_25_25_1_1_S25~RPsVFER	-1.50	1.02
CAVIN2	O95810_283_288_1_1_S288~ISSGKSsPFK	-1.48	1.20
PRPF38B	Q5VTL8_320_320_1_1_S320~sIDRGLER	-1.47	1.47
OGFR	Q9NZT2_361_382_1_1_S378~SQGDEAGGHGEDRPEPLsPKESK	-1.45	1.23
THRAP3	Q9Y2W1_248_257_1_1_S248~ERsPALKSPLQSVVVR	-1.44	1.43
XRCC4	Q13426_299_309_1_1_S304~ENSRPDSsLPETSK	-1.44	2.09
MKI67	P46013_2528_2542_1_0~AFKESPKQILDPAASVTGSR	-1.43	1.39
OGA	O60502_6_9_1_1_S6~VQKEsQATLEER	-1.42	1.51
MBLAC1	A4D2B0_56_61_1_1_S61~GPASSHREsPR*	-1.41	2.11
FAM193B	Q96PV7_6_9_1_1_S6~sRPSGGAGR*	-1.41	1.55
JPT1	Q9UK76_14_18_1_1_S14~GVDPNsRNSSR	-1.40	1.55
DST	Q03001_7559_7562_1_1_S7559~sPASKLDK	-1.40	1.28
AKAP12	Q02952_1261_1270_1_1_T1264~TEGtQEADQYADEK	-1.39	1.58
SRRM2	Q9UQ35_1690_1698_1_0~SSRSSPELTR	-1.38	1.29
PNISR	Q8TF01_203_204_1_0~ERPSSFRDR	-1.37	1.40
ATXN2L	Q8WWM7_678_684_1_1_S684~STSTPTsPGPR	-1.36	1.42
HTATSF1	O43719_385_391_1_1_S387~RSDsVSASER	-1.36	1.11
DNTTIP1	Q9H147_161_161_1_1_S161~QAEEECAHRGsPLPK	-1.35	3.03
USP14	P54578_393_394_1_0~KSSPQKEVK	-1.34	1.46
CWC22	Q9HCG8_903_903_1_1_S903~EKsPAKQK	-1.34	1.18
USP39	Q53GS9_42_46_1_1_S46~EREPEAASSRGsPVR	-1.33	1.23
MAP7D1	Q3KQU3_364_376_1_1_S366~SAsASPLTPCSVTR	-1.33	1.80
HNRNPD	Q14103_71_91_2_2_S82S83~IDASKNEEDEGHSNssPRHSEAATAQR	-1.32	1.47
CCDC124	Q96CT7_141_141_1_1_S141~VLEEGsVEAR	-1.31	1.52
LARP1	Q6PKG0_515_530_3_3_S517S521T526~ETEsAPGsPRAVtPVPTK	-1.30	1.57
TRA2A	Q13595_96_103_3_2_S98S100~SRsHsPMSNR	-1.30	1.40
DROSHA	Q9NRR4_237_237_1_1_S237~DHsHGRGER	-1.30	1.43
DPYSL3	Q14195_514_524_2_2_S518S522~GGTPAGsARGsPTRPNPPVR	-1.29	1.43
MAP1B	P46821_1396_1421_1_1_S1400~VLSPLRsPPLIGSESAYESFLSADDKASGR	-1.29	1.13
PRKDC	P78527_2620_2626_1_1_S2624~TQEGsLSAR	-1.28	1.85
NOP2	P46087_776_786_1_0~QNDTPKGPQPPTVSPIR	-1.28	1.12

DHX38	Q92620_199_199_1_1_S199~RNEPEsPR	-1.28	2.53
RPS28	P62857_21_28_1_1_S23~TGsQGQCTQVR	-1.28	1.33
SRRM2	Q9UQ35_2115_2125_1_0~MSCFSRPSMSPTPLDR	-1.27	1.41
HNRNPD	Q14103_80_91_1_0~NEEDEGHSNSSPRHSEAATAQR	-1.27	1.58
HDGF	P51858_98_103_1_0~ASGYQSSQKK	-1.27	2.14
PPP1R18	Q6NYC8_221_230_1_0~EVESRLSPGESAYQK	-1.26	1.64
CHD1L	Q86WJ1_601_607_1_1_S607~TLLEKAsQEGR	-1.26	2.18
TRPM7	Q96QT4_1248_1258_1_1_S1255~TLTAQKAsEASK	-1.26	1.44
ADD1	P35611_450_465_1_1_S464~WLNSGRGDEASEEGQNGsSPK	-1.26	1.26
MAP1B	P46821_1852_1853_1_0~DLSTPGLEK	-1.25	1.05
NUMA1	Q14980_1851_1856_1_1_S1853~ATSsTQSLAR	-1.24	1.05
UBE4B	O95155_103_112_1_0~SQSMDIDGVSCEK	-1.23	1.47
DOCK7	Q96N67_450_470_1_0~TTSGDDACNLTSFRPATLTVTNFFK	-1.22	1.48
NPM1	P06748_227_227_1_1_S227~GQEsFKKQEK	-1.22	1.25
EPS8L2	Q9H6S3_455_459_1_1_S459~QSIRNsQK	-1.22	2.21
MAP3K3	Q99759_274_275_1_1_T274~GGtYPRR	-1.21	1.13
SRSF5	Q13243_85_91_1_0~YSDRFSSR	-1.21	1.11
RSF1	Q96T23_465_475_1_1_S473~FYETKEESYsPSKDR	-1.21	1.33
CUTC	Q9NTM9_7_8_1_0~QGASSER	-1.21	1.40
LIMA1	Q9UHB6_726_726_1_1_S726~sQDVELWEGEVVK	-1.21	1.31
ZDHHC5	Q9C0B5_376_391_1_1_S380~LSRGDsLKEPTSIAESSR	-1.21	1.43
SRSF4	Q08170_420_423_1_0~SESSQREGR	-1.20	1.34
CKAP2L	Q8IYA6_635_638_1_1_S638~SCLsPKER	-1.19	1.34
INPP5J	Q15735_886_888_2_2_S886S888~HRsRsPGLAR	-1.19	1.26
SRRM2	Q9UQ35_846_866_1_0~SGTPPRQGSITSPQANEQSVTPQR	-1.18	1.28
UIMC1	Q96RL1_643_662_1_1_S653~SSETGAFRVPsPGMEEAGCSR	-1.18	1.28
ZYX	Q15942_258_270_1_1_S267~GPPASSPAPAPKFsPVTPK	-1.17	1.20
WAC	Q9BTA9_62_76_1_1_S64~RSDsPENKYSDSTGHSK	-1.17	1.20
SPANXD	Q9BXN6_44_49_1_0~TSESSTILVVR*	-1.17	1.32
TRMT6	Q9UJA5_282_291_1_1_T291~DSALVEESNGtLEEK	-1.17	1.35
ADD1	P35611_450_465_1_0~WLNSGRGDEASEEGQNGSSPK	-1.16	1.06
ACIN1	Q9UKV3_341_343_1_1_S343~FTRsQEEAR	-1.16	1.74
SRRM2	Q9UQ35_2738_2740_1_1_S2740~ETPsPRPMR	-1.16	1.61
TRA2B	P62995_201_216_1_1_T201~RPHtPTPGIYMGRPTYGSSR	-1.15	1.30
KNL1	Q8NG31_1675_1675_1_1_S1675~RCsLGIFLPR	-1.15	1.01
LARP1	Q6PKG0_766_777_2_0~SLPTTVPESPNYR	-1.15	1.40
CDC20	Q12834_41_41_1_1_S41~EAAGPAPsPMR	-1.15	1.26
NAB2	Q15742_157_162_1_1_S159~SFsPKSPLELGEK	-1.15	1.07
PPP1R9A	Q9ULJ8_94_95_1_1_S95~GKGGHSsPQRR*	-1.15	1.47
CEP170	Q5SW79_133_141_1_1_S138~SASAKsIDSK	-1.14	1.50
SRRM2	Q9UQ35_2115_2125_1_1_S2123~MSCFSRPSMsPTPLDR	-1.14	1.16
AKAP2	Q9Y2D5_839_839_1_1_S839~RKsALALR	-1.14	1.16
CLIP1	P30622_191_204_1_0~TASESISNLSEAGSIK	-1.14	1.52
MAP4	P27816_1142_1151_1_1_S1145~EAQTLDsQIQETSI	-1.13	1.51
EEF1A1	P68104_452_454_1_1_S454~VTKsAQK	-1.13	1.09
TRMT6	Q9UJA5_282_291_1_0~DSALVEESNGTLEEK	-1.11	1.10
NUCKS1	Q9H1E3_13_34_1_1_S19~VVDYSQFQEsDDADEDYGRDSGPPTKK	-1.11	1.34
ZC3HC1	Q86WB0_329_338_1_1_S329~sQDATFSPGSEQAEK	-1.11	1.80
EIF4B	P23588_488_507_1_0~SSNPPARSQSSDTEQQSPTSGGGK	-1.11	1.30
EIF4EBP1	Q13541_65_68_1_1_S65~FLMECRNsPVTK	-1.11	1.37
CDC42BPA	Q5VT25_1544_1545_1_0~RYSFRVPEEER	-1.11	1.33
SETD2	Q9BYW2_543_546_1_0~RGSSYSK	-1.10	1.19
HEATR6	Q6AI08_635_643_2_1_S635~APAGPsLEETSVSSPK	-1.10	1.27
CDC42EP1	Q00587_350_365_1_1_S350~AsWESLDEEWRAPQAGSR	-1.10	1.61
RSL1D1	O76021_392_401_1_1_S392~KsPAKSPNPSTPR	-1.10	1.05
EIF4G1	Q04637_704_704_1_1_S704~RsQQGPR	-1.09	1.25
MAP1B	P46821_1396_1421_2_2_S1396S1400~VLsPLRsPPLIGSESAYESFLSADDKASGR	-1.09	1.44
CARMIL1	Q5VZK9_1146_1151_2_2_S1150S1151~SDSKssPQAGRR	-1.09	1.10
CHMP7	Q8WUX9_429_441_1_0~LSLSEGGLVPSSKSPK	-1.08	1.70
TET2	Q6N021_69_82_1_1_S75~GSQNSRVsPDFTQESR*	-1.08	1.46
NUFIP2	Q7Z417_606_616_1_1_S608~ADTSsQGALVFLSK	-1.08	1.08
LIMA1	Q9UHB6_581_583_2_2_S582S583~RSssLKER	-1.08	1.11
ZC3HC1	Q86WB0_329_350_3_1_S344~SQDATFSPGSEQAEKsPGPIVSR	-1.07	1.57
SAFB	Q15424_614_621_1_1_S617~KSRDsESHSR	-1.07	1.67
CPSF7	Q8N684_427_431_2_2_S427S429~ERsPsRSR	-1.07	1.05
RBM28	Q9NW13_712_717_1_0~QQLSSEQVSR	-1.06	1.19
MARCKSL1	P49006_148_151_1_1_S151~AAATPEsQEPQAK	-1.06	1.72
ANK2	Q01484_29_34_1_1_S34~SDSNAsFLR	-1.06	1.92
TRAFD1	O14545_409_415_2_2_T414S415~LDSQPQEtsPELPR	-1.05	1.07
MKI67	P46013_1736_1751_1_0~VSYRASQPDLVDTPTSSKPQPK	-1.05	1.18
LIMA1	Q9UHB6_617_625_1_1_S619~KGWSMsEQSEESVGGR	-1.04	1.21
TMX1	Q9H3N1_270_280_1_0~QRSLGPSLATDKS	-1.04	1.50
AFAP1L1	Q8TED9_745_751_1_1_S747~RSPsIVASNQGR	-1.04	1.11
SRSF4	Q08170_431_444_2_2_S442S444~GESENAGTNQETRsRsR	-1.04	1.58
GBF1	Q92538_1778_1791_2_2_S1784S1789~AASSSSPGsPVASsPSR	-1.04	1.47
PDLIM5	Q96HC4_309_323_1_0~KANNSQEPSPQLASSVASTR	-1.03	1.47
CENPC	Q03188_167_177_1_1_S177~TSVSQNVIPSsAQK	-1.02	1.66

NPM1	P06748_195_199_1_1_T199~SIRDtPAKNAQK	-1.02	1.38
APC	P25054_1031_1044_1_1_S1042~YSDEQLNSGRQsPSQNER	-1.02	1.09
FOSL2	P15408_194_200_1_1_S200~ISPEERRsPPAPGLQPMR	-1.02	1.49
SRRM2	Q9UQ35_525_527_2_2_S525S527~WGRsRsPQR	-1.01	1.15
LARP1	Q6PKG0_526_536_1_0~AVTPVPTKTEEVSNLK	-1.01	1.83
ZNF185	O15231_531_534_1_1_S531~REsCTSR	-1.01	1.10
SUPT16H	Q9Y5B9_1023_1027_1_1_S1023~KAsVHSSGR	-1.01	1.01
PEX19	P40855_66_66_1_1_S66~DALFAsQEK	-1.01	1.87
SAFB	Q15424_601_604_1_0~SVVSFDKVKEPR	-1.01	2.28
PPP1R3D	O95685_74_78_2_0~SLPSSPERR	-1.00	1.35
SP4	Q02446_118_140_1_1_S136~ENNVSQPASSSSSSSSSNNGSAsPTKTK	-1.00	1.37
SH3GL1	Q99961_286_288_1_0~IAASSSFR	-1.00	1.05
NIPBL	Q6KC79_2663_2672_1_1_T2667~NNTAAEtEDDESDGEDR	1.01	1.13
GEMIN5	Q8TEQ6_757_778_1_1_S778~LESIDGNEEESMKENSGPVENGVsDQEGEEQAR	1.02	1.26
ZC3H18	Q86VM9_32_34_1_1_S34~DSGsDQDLDGAGVR	1.02	1.31
ZBTB7A	O95365_549_549_1_1_S549~HFKDEDEDEDVAsPDGLGR	1.03	2.84
ZBTB7A	O95365_337_341_1_1_S337~AGAAAGDsDEESRADDK	1.03	3.06
ZC3H18	Q86VM9_59_74_1_1_S74~GPSQEEEDNHSDEEDRAsEPK	1.03	1.40
HNRNPC	P07910_299_306_1_1_S306~EAEEGEDDRDSANGEDDs	1.03	1.06
SALL2	Q9Y467_802_820_2_2_S802S806~GDsEEAsGAEEEVGTVAAAATAGK*	1.03	1.47
ESF1	Q9H501_657_671_1_1_S657~ALAEEAsEEELPSDVDLNDPYFAEEVK	1.04	1.30
IWS1	Q96ST2_300_304_1_1_S300~VsDSESEGPQK	1.05	1.24
NES	P48681_1003_1016_1_1_S1016~EEVVEQGELNATEEVWIPGEGHPEsPEPK	1.05	1.31
MAX	P61244_129_151_1_1_S144~GSTISAFDGGSDSSSEsEPEEPQSR	1.05	1.69
ALKBH5	Q6P6C2_60_71_1_1_S64~YQEDsDPERSDYEEQQLQK	1.06	1.54
HNRNPC	P07910_299_306_1_0~EAEEGEDDRDSANGEDDS	1.06	1.09
THAP12	O43422_132_156_1_1_S141~ETNNSNAQNPsEEEGEGQDEDILPLTLEEK	1.07	2.25
SAP30BP	Q9UHR5_72_79_1_0~QSEDDDSETEKPEADDPK	1.08	1.42
TOP2B	Q02880_1457_1478_1_1_S1466~SEDDSAKFDsNEEDSASVFSPSFGLK	1.08	2.02
ZP2	Q05996_217_248_2_0~MTFHVPFNATGVTHYVQGNSHLYMVSLKLTFISPGQK	1.10	1.10
TRA2B	P62995_37_43_2_2_S37S39~sRsKEDSR	1.10	1.03
GLDC	P23378_888_910_1_1_T902~LQDYGFHAPTMSWPVAGtLMVEPTESEDKAELDR*	1.11	1.38
EEF1D	P29692_147_162_1_1_S162~KPATPAEDDEDDDIDLFGsDNEEEDKEAAQLR	1.11	1.06
HTATSF1	O43719_642_650_1_1_S642~VFDDEsDEKEDEEYADEK	1.14	1.35
NUDC	Q9Y266_136_145_1_0~NGSLDSPGKQDTEEDEEEDEKDK	1.16	1.22
HSP90AA1	P07900_252_263_1_1_S263~ESEDKPEIEDVGsDEEEEKK	1.18	1.23
TIAM2	Q8IVF5_1565_1569_1_1_S1569~ESDILsDEDDDHR*	1.19	1.59
SUB1	P53999_9_19_2_2_S17S19~ELVSSSSSGSDsDsEVDKK	1.20	2.52
SUB1	P53999_9_19_2_0~ELVSSSSSGSDSDSEVDKK	1.20	2.86
TRMT10A	Q8TBZ6_318_318_1_1_S318~NELDsPHEEK	1.21	1.14
AKAP12	Q02952_640_651_2_2_S644S645~SATLssTESTASEMQEEMK	1.22	1.98
IWS1	Q96ST2_235_237_1_1_S235~HQAsDSENEELPKPR	1.23	1.47
MAP1B	P46821_1016_1016_1_1_S1016~GEAEQsEEEADEEDKAEDAR	1.23	1.35
ERICH1	Q86X53_254_259_1_1_S254~QEEGADAsEEDPTPAGEEDVK	1.24	1.18
GEMIN5	Q8TEQ6_757_778_1_0~LESIDGNEEESMKENSGPVENGVSDQEGEEQAR	1.26	1.26
TCEAL4	Q96EI5_82_88_1_1_S88~EGESEMEGGsER	1.27	4.17
CEP170	Q5SW79_497_511_1_1_S497~DNDDDQsDKGTYTIELENPNSEEVEAR	1.29	2.71
SEC31A	O94979_521_532_1_0~DSDQVAQSDGEESPAAEEQLLGEHIK	1.31	1.64
TRA2A	Q13595_20_24_1_1_S20~sPTGTPAR	1.32	1.06
MAP1B	P46821_970_977_1_0~HSPTEDEESAK	1.34	1.28
ZC3H18	Q86VM9_162_162_1_1_T162~AGAEDDEEKGEGtPREEGK	1.34	1.80
G3BP2	Q9UN86_163_204_1_0~QPSPEPVQENANSGYYEAHPVTNGIEEPLEESSHEPEPEPESETK	1.40	1.97
ANKRD11	Q6UB99_752_752_1_1_S752~EKsPKEEK	1.46	2.24
SUB1	P53999_9_19_1_1_S19~ELVSSSSSGSDSDsEVDKK	1.48	3.27
HSP90AB1	P08238_226_226_1_1_S226~EKEIsDDEAEEEK	1.76	5.89
